# Trace metals health risk appraisal in fish species of Arabian Sea

**DOI:** 10.1186/s40064-016-2436-6

**Published:** 2016-06-24

**Authors:** Kousar Yasmeen, Muhammad Aslam Mirza, Namra A. Khan, Nazish Kausar, Atta-ur Rehman, Muddasir Hanif

**Affiliations:** Department of Chemistry, Federal Urdu University of Arts, Science and Technology, Gulshan-e-Iqbal Campus, Karachi, 75300 Pakistan; Department of Chemistry, Mirpur University of Science & Technology (MUST), Mirpur, 10250 AJ&K Pakistan; Nabiqasim Industries (Pvt) Ltd. Commerce Centre, Hasrat Mohani Road, Karachi, 74200 Pakistan

**Keywords:** Environmental pollution, Fish species, Trace metals, Karachi, Human health

## Abstract

Fish is a vital food for humans and many animals. We report an environmental monitoring study to assess the trace metals in fish species caught from Arabian Sea and commercially available in the coastal city Karachi, Pakistan. Heavy metals such as copper, iron, lead and cadmium were determined in the skin, fillet and heart of the fish species *Pampus argenteus*, *Epinephelus chlorostigma*, *Rachycentron canadum*, *Scomberomorus commerson*, *Johnius belangerii*, *Labeo rohita*, *Lutjanus argentimaculatus*, *Trachinotus blochii*, *Pomadsys olivaceum* and *Acanthopagrus berda* by the atomic absorption spectrophotometer. The concentration (mg kg^−1^, dry weight) range was: Cd (0.00–0.041), Cu (0.006–0.189), Fe (0.413–4.952) and Pb (0.00–0.569). Cadmium, copper and iron levels were below the tolerable limits whereas concentration of lead in the skins of *S. commerson*, *E. chlorostigma*, *J. belangerii*, *A. berda*; *L. argentimaculatus*, fillets of *J. belangerii*, *E. chlorostigma* and in the heart of *J. belangerii* exceeded the recommended limits. Therefore fish skin should be discouraged as food for humans or animals. The results indicate that a number of fish species have higher concentration of heavy metals dangerous for human health. Since the fish *P. olivaceum* (Dhotar) has the lowest level of trace metals therefore we recommend it for breeding and human consumption.

## Background

Oceans are a great source of foodstuff for humans and cover 70 % of the earth’s surface. Seafood, particularly fish is used by a significant portion of the world’s population. Fish is a recommended food for the adequate human health. It is a vital source of protein and healthy long chain omega-3 fats capable to prevent heart diseases and improves heart vessel functions. Seafood or fish is also rich with minerals and vitamins which are considered to be important for maintaining human health. However this natural food is being poisoned due to the increment of heavy metals. The reason for this increment is critical environmental pollution. The concentration of heavy metal contamination is an important factor because of its toxic effects on human health (Shahid-ul-Islam and Tanaka 2004; Zehra et al. 2003). These trace metals are tolerable at extremely low concentration and above certain concentration they become toxic for humans. The major risk to human health is disclosure to cadmium, lead, mercury and arsenic (Alam et al. 2002; Maffucci et al. 2005). Heavy metal contamination of marine animals and aquatic ecosystem is associated with a wide range of sources like dumping of industrial waste, untreated sewage, spills of toxic chemicals, agricultural chemicals and others, which can impact the health of marine life. The heavy metals easily gather in the tissues of aquatic life forms and transferred to humans through consumption of contaminated food (Ahmed 1977; Olowu et al. 2010; Qadir et al. 2011; Tuzen 2002).

Since fish contains important nutrients therefore around the world, research has been conducted to document trace metal concentration in different types of marine and fresh water fish species. We present a study of heavy metals concentration like iron, copper, cadmium and lead in the edible and commercially important fish species commonly used by the people in Karachi, Pakistan. It is well known that metals play an important role in many kinds of biological functions. For example, Cu, Mn, Zn, and Fe are vital micronutrients, but become noxious at concentrations greater than those essential for typical growth (Nies 1999) While Cd, Hg, As and Pb are toxic metals even at very low levels (Nies 1999; Wood 1974).

Pollution is one of the global problems. It has different types such as air, soil, water; noise, light, visual and radioactivity etc. (Hameed et al. 2012). There is main focus on the marine pollution because it is a serious issue across the globe (Khoshnood et al. 2012). The estimated population of coastal city Karachi is over 17 million and it is the biggest center of economic activity in Pakistan, with about 6000 small and large industrial units (Qureshi and Riffat 2010). Karachi is situated on the northern boundary of the Arabian Sea. The coastal zone of Karachi covers about 135 km area (Mashiatullah et al. 2012). The Port is located close to the Karachi and the city is famous for the industrial development, education and multicultural activities (Khattak et al. 2012). The disposal of industrial and municipal waste produced from the city is 292 million gallons per day clumped into the sea in the course of two major drains known as Malir and Lyari River. Fishing industry plays an important role in the economy of Pakistan. Most of the population of the coastal areas of Sindh and Balochistan depend on fisheries for the daily food and living. It is also an important source of exports and foreign exchange. Inside Pakistan, fishing is done from rivers, dams, lakes, ponds and canals. Nearly 41 % of the total fish production and packaging obtained from Inland resources.

## Experimental

### Sampling area

The coastal area of Pakistan limits Arabian Sea. It is around 960 km long and it stretches from the Rann of Kutch South East to the Gawadar North West. The territorial coastal zone of Pakistan consists of 23,820 km^2^ while Exclusive Economic Zone (EEZ) of Pakistan is about 240,000 km^2^. Fishermen routinely go for fishing from Pakistani coastal areas and supply sea food back to the different markets of Karachi.

Ten different commercially important and commonly consumed edible marine fish species were collected from different markets of Karachi. *Epinephelus chlorostigma* locally called Gisser, *Labeo rohita* (Rohu), *Pampus argenteus* (White Pomfret), *Rachycentron canadum* (Sangra), *Scombermous commerson* (Surmai), *Johnius belangerii* (Mushka), *Lutjanus argentimaculatus* (Hira), *Trachinotus blochii* (Sonaf), *Pomadasys olivaceum* (Dhotar) and *Acanthopagrus berda* (Dandya) were randomly selected and purchased from five different markets of Karachi, Liaquat Market (Malir), Water Pump (F. B. Area), New Shabuddin (Lines Area, Saddar), Bangali Parah (Korangi Creek), Sabzi Market (Shah Faisal Colony), during July–October, 2013.

### Sample preparation

**Fig. 1:**
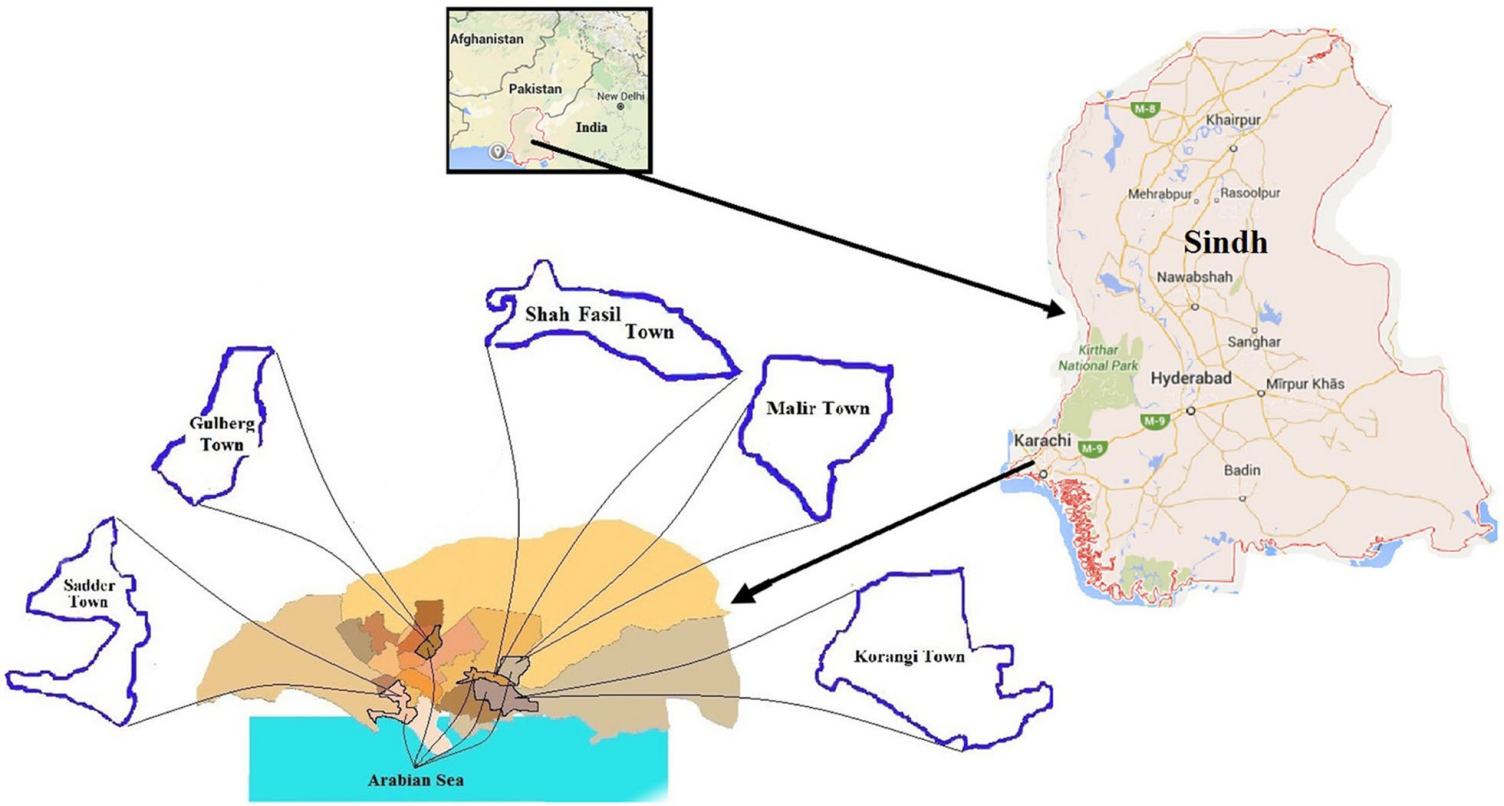
Map showing sampling sites of five fish species

Three specimens of each fish species were purchased from five markets (Fig. 1) and then separately placed in high quality transparent polythene bags and preserved in deep-freezer until dissection. The fish samples were defrosted, by washing them with de-ionized water. After washing, fish samples were dissected to obtain different parts such as heart, skin and fillet. Each part was separately washed with de-ionized water for several minutes. Samples were dried at 100 °C for 48 h and weighed. The dried samples of each species were ground to fine particles using mortar and pestle. Wet digestion of different parts of each specie was carried out by taking 3 g of powdered sample of skin and fillet while heart of each species as its whole weight in nitric acid (65 %) and hydrogen peroxide as a decolorizing agent. The mixture of each tissue was refluxed on a hot plate until fumes ceased to evolve. Then the mixture was filtered and the filtrate was diluted to 100 mL with de-ionized water in a volumetric flask (Skoog et al. 1996). These solutions were taken in bottles and subjected to analysis.

### Heavy metals analysis

Heavy metals for instance cadmium, copper, iron and lead concentration in fish samples (heart, skin and fillet) were measured by using AAS (Perkin Elmer AAnalyst 700). Standards of cadmium, copper, iron and lead were prepared from the respective stock solutions (Merck). 1 % HNO_3_ was used as a blank which was prepared in deionized water. Limit of detection was determined by using absorbance of blank and the standard solution of 1 ppm of each element. The limit of detection for Cd, Cu, Fe and Pb were 11.568, 21.452, 3.365 and 1.743 (µg kg^−1^) respectively. The parameters set for analysis on AAS were:

**Table:** 

Metal	Flame type	Slit width (nm)	Wavelength (nm)	Lamp
Cadmium	Air-acetylene	0.7	288.8	EDL
Copper	Air-acetylene	0.7	324.8	HCL
Iron	Air-acetylene	0.2	248.3	HCL
Lead	Air-acetylene	0.7	283.3	EDL

ANOVA (One way, unstaked) at 95 % confidence level is used to compare results of fish species

## Results

**Fig. 2:**
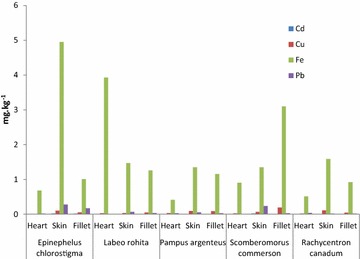
Mean concentration of metals in five fish species

**Fig. 3:**
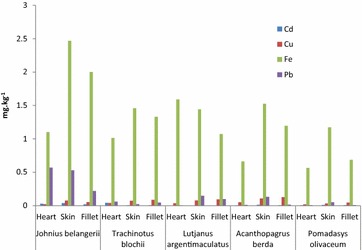
Mean concentration of metals in five fish species

 The results of study showed that the concentration of Cd in all fish species were in range of 0.00–0.041 mg kg^−1^ (heart), 0.00–0.037 mg kg^−1^ (skin) and 0.00–0.020 mg kg^−1^ (fillet). Copper levels in the heart, skin and fillet were measured as 0.006–0.052, 0.030–0.108 and 0.042–0.189 mg kg^−1^ respectively. Analyzed Iron content in heart tissues was 0.413–3.931 mg kg^−1^, skin 1.173–4.952 mg kg^−1^ and fillet 0.685–3.098 mg kg^−1^. Lead concentration (mg kg^−1^) was found to be 0.00–0.569 (heart), 0.012–0.529 (skin) and fillet 0.008–0.218 (Table 1; Figs. 2, 3).

**Table 1:** Metals concentration (mg kg^−1^) in samples of ten (Arabian Sea Origin) fish species

Fish species	Muscle	Metals			
		Cd Mean ± SD	Cu Mean ± SD	Fe Mean ± SD	Pb Mean ± SD
*Epinephelus chlorostigma*	Heart	0.002 ± 0.0007^bc^	0.006 ± 0.004^b^	0.679 ± 0.042^b^	0.014 ± 0.012^b^
	Skin	0.019 ± 0.005^a^	0.099 ± 0.066^a^	4.952 ± 1.824^a^	0.276 ± 0.260^a^
	Fillet	0.015 ± 0.012^ab^	0.051 ± 0.024^ab^	1.006 ± 0.912^b^	0.168 ± 0.155^ab^
*Labeo rohita*	Heart	BDL^c^	0.024 ± 0.00^ab^	3.931 ± 0.033^a^	BDL^b^
	Skin	0.005 ± 0.004^bc^	0.030 ± 0.016^ab^	1.469 ± 1.236^b^	0.066 ± 0.058^ab^
	Fillet	0.003 ± 0.001^bc^	0.048 ± 0.001^ab^	1.259 ± 0.531^b^	0.030 ± 0.018^b^
*Pampus argenteus*	Heart	0.002 ± 0.002^bc^	0.030 ± 0.016^ab^	0.413 ± 0.140^b^	0.027 ± 0.024^b^
	Skin	0.005 ± 0.003^bc^	0.091 ± 0.025^a^	1.349 ± 0.230^b^	0.051 ± 0.020^ab^
	Fillet	0.004 ± 0.003^bc^	0.084 ± 0.013^a^	1.157 ± 0.170^b^	0.027 ± 0.017^b^
*Scomberomorus commerson*	Heart	BDL^c^	0.021 ± 0.028^a^	0.903 ± 0.313^b^	BDL^b^
	Skin	0.019 ± 0.008^a^	0.067 ± 0.010^a^	1.347 ± 0.173^b^	0.236 ± 0.039^ab^
	Fillet	0.002 ± 0.00^bc^	0.189 ± 0.045^a^	3.098 ± 0.117^b^	0.024 ± 0.011^b^
*Rachycentron canadum*	Heart	0.005 ± 0.003^bc^	0.015 ± 0.016^a^	0.508 ± 0.140^b^	0.036 ± 0.022^ab^
	Skin	0.002 ± 0.001^bc^	0.108 ± 0.021^a^	1.586 ± 0.230^b^	0.012 ± 0.005^b^
	Fillet	0.007 ± 0.004^abc^	0.042 ± 0.004^a^	0.922 ± 0.657^b^	0.013 ± 0.003^b^
*Johnius belangerii*	Heart	0.027 ± 0.0007^c^	0.021 ± 0.0168	1.101 ± 0.0117^ab^	0.569 ± 0.0066^a^
	Skin	0.037 ± 0.034^b^	0.075 ± 0.010^abc^	2.468 ± 0.726^a^	0.529 ± 0.451^a^
	Fillet	0.020 ± 0.014^a^	0.053 ± 0.004^bc^	2.001 ± 0.950^ab^	0.218 ± 0.108^ab^
*Trachinotus blochii*	Heart	0.041 ± 0.005^bc^	0.038 ± 0.018^bc^	1.013 ± 0.196^ab^	0.059 ± 0.042^b^
	Skin	0.004 ± 0.001	0.074 ± 0.016^abc^	1.457 ± 0.037^ab^	0.021 ± 0.018^b^
	Fillet	0.004 ± 0.001	0.088 ± 0.024^abc^	1.330 ± 1.101^ab^	0.046 ± 0.025^b^
*Lutjanus argentimaculatus*	Heart	BDL	0.035 ± 0.022^c^	1.589 ± 0.941^ab^	0.001 ± 0.001^b^
	Skin	BDL	0.078 ± 0.049^abc^	1.442 ± 0.186^ab^	0.147 ± 0.099^b^
	Fillet	BDL	0.092 ± 0.029^abc^	1.071 ± 0.126^ab^	0.099 ± 0.034^b^
*Acanthopagrus berda*	Heart	0.001 ± 0.001	0.052 ± 0.019^bc^	0.66 ± 0.044^b^	0.0087 ± 0.002^b^
	Skin	0.012 ± 0.002	0.107 ± 0.019^ab^	1.526 ± 0.149^ab^	0.131 ± 0.028^b^
	Fillet	0.004 ± 0.003	0.127 ± 0.036^a^	1.196 ± 0.262^ab^	0.015 ± 0.007^b^
*Pomadasys olivaceum*	Heart	0.004 ± 0.001	0.019 ± 0.018	0.564 ± 0.224^b^	0.005 ± 0.004^b^
	Skin	0.009 ± 0.005	0.032 ± 0.015^c^	1.173 ± 0.775^ab^	0.051 ± 0.038^b^
	Fillet	0.006 ± 0.001	0.045 ± 0.015^bc^	0.685 ± 0.090^ab^	0.008 ± 0.006^b^
Reference values	IAEA-407 (2003)	0.189	3.280	146.0	0.120
	WHO (1989)	DNA	30.0	100	2.0
	FAO (1983)	DNA	30.0	DNA	0.50

Data generated from individual tripliocate readings (n = 3)

*BDL* below detection limit, *DNA* data is not available

^a–c^ Values indicates that means (X) that do not share a letter are significantly different at 95 % confidence level (ANNOVA, One way, unstaked)

*Epinephelus chlorostigma* (Gisser) was found to have higher accumulation of metals in the skin. Metal contents in different parts were in order of skin > fillet > heart. Iron was highest (4.952 mg kg^−1^) in the skin while concentrations of Pb, Cu and Cd in the skin (mg kg^−1^) were 0.276, 0.099 and 0.019 respectively. A comparison of concentrations with those set by the International Atomic Energy Agency-407 limits (International Atomic Energy Agency-407 2003) for the edible fish showed higher concentration of lead in the skin (0.276 mg kg^−1^) and fillet (0.168 mg kg^−1^) of *E. chlorostigma*. Metal accumulation in the fish *E. chlorostigma* showed the following order iron > lead > copper > cadmium. Varying accretion pattern of heavy metals was analyzed in the different parts of *L. rohita* (Rohu). Heart of *L. rohita* contains high concentration of iron (3.931 mg kg^−1^), cadmium and lead were higher in the skin and copper in the fillet. Order of metal content in fish *L. rohita* was iron > copper > lead > cadmium.

The skin of *P. argenteus* was found to accumulate higher amounts of all the investigated heavy metals. Iron showed excess in all parts (2.919 mg kg^−1^). The contents of heavy metals in *P. argenteus* (White Pomfret) showed the following order iron > copper > lead > cadmium. Iron was found higher in the heart, skin and fillet of *Scomberomorus commerson* (Surmai) while highest concentration of cadmium 0.019 mg kg^−1^ was present in the skin. Skin of *S. commerson* also contained high concentration of lead (0.236 mg kg^−1^) and exceeded the International Atomic Energy Agency-407 limits (Table 1). While copper was high in the fillet (0.189 mg kg^−1^). Accumulation order of heavy metals in *S. commerson* was iron > copper > lead > cadmium. Like other fish species, *R. canadum* also accumulated high amounts of iron in its heart, skin and fillet. Concentrations of metals such as cadmium, copper and lead were 0.007 mg kg^−1^ (fillet), 0.108 mg kg^−1^ (skin) and 0.036 mg kg^−1^ (heart) respectively. Heavy metal contents in *R. canadum* (Sangra) were in the following order iron > copper > lead > cadmium.

The concentration (mg kg^−1^) of cadmium, copper, iron and lead in *J. belangerii* (Mushka) was in the order: heart: iron > lead > cadmium > copper; skin: iron > lead > copper > cadmium and in fillet: iron > lead > copper > cadmium. The highest and lowest amounts of iron and cadmium were found in the skin and fillet except heart in which copper was the least. The total metal contents were in the order of iron > lead > copper > cadmium. Cadmium, copper and iron accumulation were high in skin; the concentration of lead was high in the heart of *J. belangerii*. Lead in heart (0.569 mg kg^−1^) and skin (0.529 mg kg^−1^) exceeded the IAEA-407 and FAO limits. In fish species *T. blochii* (Sonaf), Cd (0.041 mg kg^−1^) and Pb (0.059 mg kg^−1^) accumulations were higher in the heart; the concentration of Cu was higher in the fillet (0.088 mg kg^−1^), while concentration of Fe (1.457 mg kg^−1^) was higher in the skin. The accrual order of metals was iron > copper > lead > cadmium.

The concentrations (mg kg^−1^) of cadmium, copper, iron and lead in the heart, skin and fillet of the fish *L. argentimaculatus* (Hira) were in the order of Heart: iron > copper > lead; Skin: iron > lead > copper; Fillet: iron > lead > copper and cadmium not detected in all tissues. The highest accumulation of iron (1.589 mg kg^−1^) was found in the heart, copper in fillet (0.092 mg kg^−1^) and lead in skin (0.147 mg kg^−1^).

Table 2 shows that in *A. berda* (Dandya) maximum level of cadmium accumulated in skin (0.012 mg kg^−1^), copper in fillet (0.127 mg kg^−1^), iron (1.526 mg kg^−1^) and lead (0.131 mg kg^−1^) in skin. The concentrations (mg kg^−1^) of cadmium, copper, iron and lead in *A. berda* were iron > copper > lead > cadmium. The concentrations of cadmium, copper, iron and lead in *Pomadsys olivaceum* (Dhotar) heart, skin and fillet was in the order of Heart: iron > copper > lead > cadmium; Skin: iron > lead > copper > cadmium and Fillet: iron > copper > lead > cadmium. This indicates similar accumulation pattern of metals in these species of fish. The highest amount of iron was in the skin (1.173 mg kg^−1^) and least amount of cadmium was found in the heart (0.004 mg kg^−1^).

**Table 2:** Comparison of metals in fish species with other studies (mg kg^−1^)

Location	Fish species	Cd	Cu	Fe	Pb	Reference #
Keti Bunder Thatta, Pakistan	*Pampus argenteus*	0.024	0.002	0.081	3.60	18
Keti Bunder Thatta, Pakistan	*Labeo rohita*	0.035	0.001	0.247	0.133	18
Indus River, Pakistan	*Oreochromis mossambicus*	–	–	–	2.87–3.60	26
Indus River, Pakistan	*Cyprinus carpio*	–	–	–	1.08–1.52	27
Baluchistan coast, Pakistan	*Acanthopagrus berda*	0.076	0.416	–	0.326	2
Arabian sea, Pakistan		0.320	–	–	4.120	19
North side of Hormoz striate, Persian Gulf and Iran		0.205	–	–	1.055	12
Osaka, Japan		0.027	–	–	0.048	20
Manila Bay, Philippines		0.024	–	–	0.135	21
Masan Bay, Korea		0.015	–	–	0.076	22

## Discussion

Cadmium is a noxious element that enhances the formation of kidney stones and excretion of calcium in urine. Skeletal damage may be caused by long term exposure to cadmium (Jarup 2003). Results indicate that the highest accretion of cadmium (0.041 mg kg^−1^) is found in the heart of *T. blochii*. Previous studies have reported that in different fish species (Table 2), Cd was (0.024–0.035 mg kg^−1^) in Keti Bunder Thatta, (0.076 mg kg^−1^) in Baluchistan coast (Tabinda et al. 2010) and (0.320 mg kg^−1^) in the Arabian Sea (Tariq et al. 1994). The levels of Cd were higher than the other regions reported data (Osaka, Japan 0.027 mg kg^−1^, Manila Bay, Philippines 0.024 mg kg^−1^ and Masan Bay, Korea 0.015 mg kg^−1^) (Masahiro et al. 1999; Maricar et al. 1997; Kwon and Lee 2001). Cadmium in the fish species samples were in the order of *J. belangerii* > *T. blochii* > *E. chlorostigma* > *S. commerson* > *P. olivaceum* > *A. berda* > *R. canadum* > *P. argenteus* > *L. rohita*. Cadmium is not detected in the skin, fillet and heart of *L. argentimaculatus*, therefore, it is considered Cd-free fish.

Cu is vital for the apt functioning of various organs and metabolic course of actions. It is a micro nutrient that is incorporated into a variety of proteins and metal conenzymes performing important metabolic functions, necessary for growth and development of bones, for the maintenance of health, brain, heart, connective tissues and many other organs present in human body. Variable concentration of Cu was recorded in the muscles, skin and heart of all species of fish. High accumulation of copper (0.189 mg kg^−1^) was found in Fillet of *S. commerson*. Cu levels were below the permissible limit (30 mg kg^−1^) of WHO and FAO (World Health Organization 1989; Food and Agricultural Organization 1983). Cu concentrations in reported data were 0.002 mg kg^−1^ in *P. argenteus*, 0.001 mg kg^−1^ in *L. rohita* from Keti Bunder Thatta, Pakistan (Tabinda et al. 2010) and 0.416 mg kg^−1^ from Baluchistan coast, Pakistan (Zehra et al. 2003). Accumulation of copper was found in the following order; *A. berda* > *S. commerson* > *L. argentimaculatus* = *P. argenteus* > *T. blochii* > *R. canadum* > *E. chlorostigma* > *J. belangerii* > *L. rohita* > *P. olivaceum*.

Major role of Fe is to merge with protein and copper in the formation of hemoglobin. Iron deficiency results in anemia. Iron accumulation was found higher in the fillet of *S. commerson* (3.098 mg kg^−1^), in the skin of *E. chlorostigma* (4.952 mg kg^−1^) and in the heart of *L. rohita* (3.931 mg kg^−1^) fish species. In fish species *P. argenteus* and *L. rohita* from Keti Bunder Thatta, Pakistan, Fe was 0.081 and 0.247 mg kg^−1^ respectively (Jarup 2003). The acceptable level of Fe in fish is 100 mg kg^−1^ (World Health Organization 1989). Accumulation of iron in the ten fish species showed the following order *L. rohita* > *E. chlorostigma* > *J. belangerii* > *S. commerson* > *L. argentimaculatus* > *T. blochii* > *A. berda* > *R. canadum* > *P. argenteus* > *P. olivaceum*.

Pb is a non-essential and toxic element investigated through biological activities (Adeyeye et al. 1996). The rapid industrialization has augmented the risk of lead exposure to humans via diverse kinds of food chains. The lead exposure may show terrible effects on the organs particularly kidneys, blood circulatory system and nervous system of a person. Lead growth was found high in the skin of *S. commerson* (0.236 mg kg^−1^), *E. chlorostigma* (0.276 mg kg^−1^), *A. berda* (0.131 mg kg^−1^), *L. argentimaculatus* (0.147 mg kg^−1^); in fillet of *J. belangerii* (0.218 mg kg^−1^), *E. chlorostigma* (0.168 mg kg^−1^) and crossed the recommended value of IAEA-407 while IAEA-407 and FAO recommended values were exceeded by skin (0.529 mg kg^−1^) and heart (0.569 mg kg^−1^) of *J. belangerii*. Pb levels were lower than the previously reported studies of Tabinda et al. 2010 (3.60 mg kg^−1^, Keti Bunder Thatta, Pakistan) Jabeen and Chaudhry 2009 (2.87–3.60 mg kg^−1^, Indus River, Pakistan) (Jabeen and Chaudhry 2009); Jabeen and Chaudhry 2010 (1.08–1.52 mg kg^−1^, Indus River, Pakistan) (Jabeen and Chaudhry 2010); Tariq et al. 1994 (4.120 mg kg^−1^, Arabian **S**ea, Pakistan) (Tariq et al. 1994) and Khoshnood et al. 2012 (1.055 mg kg^−1^, Persian Gulf, Iran) (Khoshnood et al. 2012). Lead accumulated in the 10 fish species showed the following order *J. belangerii* > *E. chlorostigma* > *S. commerson* > *L. argentimaculatus* > *A. berda* > *T. blochii* > *P. argenteus* > *L. rohita* > *P. olivaceum* > *R. canadum*. The permissible limit of lead is 0.120 mg kg^−1^ (IAEA-407 2003), 2.0 mg kg^−1^ (WHO 1989) and 0.5 mg kg^−1^ (FAO 1983).

## Conclusion

This work gives information regarding the concentration of heavy metals present in the edible samples of fish taken from the markets in Karachi. Iron and copper are essential while cadmium and lead are toxic for humans. Heavy metal concentrations recorded in fish species were compared with FAO, WHO and the International Atomic Energy Agency-407. It was noted that the concentration of Pb in the skin of *S. commerson* (Surmai), *E. chlorostigma* (Gisser), *A. berda* (Dandya) and *L. argentimaculatus* (Hira); fillet of *J. belangerii* (Mushka) and *E. chlorostigma* exceeded the IAEA-407 recommended values whereas skin and heart of *J. belangerii* crossed the permissible limits of IAEA-407. Iron, cadmium and Copper concentrations in all samples were within the acceptable limits of AEA-407, FAO and WHO. The results showed that the lead concentration exceeded the tolerable values in some fish samples. Lead is a toxic metal and it possesses hazardous effects on humans so consumption of fish contaminated with these metals can be a health risk. Therefore, it is essential that biological screening of the fish and water should be done frequently to make sure the unremitting safety of the seafood particularly fish. Secure removal into the sea should be practiced to avoid possible contamination especially heavy metals into the food-chain. Due to low concentration of toxic metals, fish species such as *P. olivaceum*, *P. argenteus* and *R. canadum* are recommended as sea food.
